# Supplementary material for: Association between oral microbiome and seven types of cancers in East Asian population: a two-sample Mendelian randomization analysis
Source: Front Mol Biosci. 2023 Nov 21;10:1327893. doi: 10.3389/fmolb.2023.1327893 (PMC10702768; doi:10.3389/fmolb.2023.1327893)
Supplement: Supplementary file 1 [file DataSheet1.ZIP › Supplementary Materials/MR plots of saliva/saliva═╝/ovarian cancer/pheno.858_to_ovarian cancer_leave_one_out.pdf]

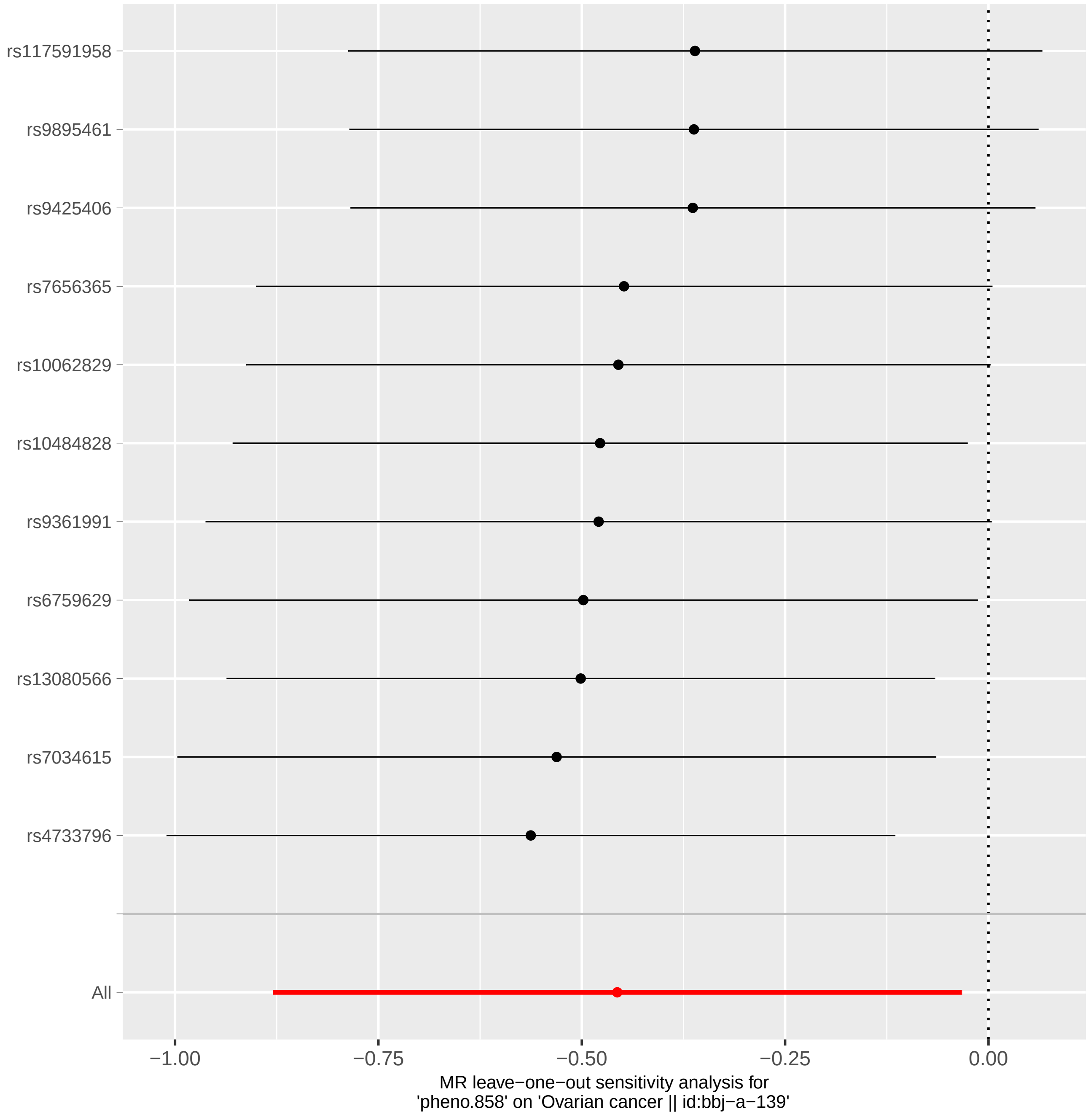
rs117591958
rs9895461
rs9425406
rs7656365
rs10062829
rs10484828
rs9361991
rs6759629
rs13080566
rs7034615
rs4733796
All
−1.00
−0.75
−0.50
−0.25
0.00
MR leave−one−out sensitivity analysis for
'pheno.858' on 'Ovarian cancer || id:bbj−a−139'
